# Experimental-analytical approach to assessing mechanosensitive cartilage blood marker kinetics in healthy adults: dose-response relationship and interrelationship of nine candidate markers

**DOI:** 10.12688/f1000research.52159.2

**Published:** 2022-01-11

**Authors:** Simon Herger, Werner Vach, Anna-Maria Liphardt, Corina Nüesch, Christian Egloff, Annegret Mündermann

**Affiliations:** 1 Department of Orthopaedics and Traumatology, University Hospital Basel, Basel, BS, 4031, Switzerland; 2 Department of Spine Surgery, University Hospital Basel, Basel, BS, 4031, Switzerland; 3 Department of Biomedical Engineering, University of Basel, Allschwil, BL, 4123, Switzerland; 4 Department of Clinical Research, University of Basel, Basel, BS, 4031, Switzerland; 5 Basel Academy for Quality and Research in Medicine, Basel, Switzerland; 6 Department of Internal Medicine 3 – Rheumatology and Immunology, Friedrich-Alexander-University Erlangen-Nuremberg (FAU), Universitätsklinikum Erlangen, Erlangen, Germany

**Keywords:** cartilage biomarkers, walking stress test, cartilage mechanosensitivity, mechanical loading, dose-response relationship

## Abstract

**Purpose:** To determine the suitability of selected blood biomarkers of articular cartilage as mechanosensitive markers and to investigate the dose-response relationship between ambulatory load magnitude and marker kinetics in response to load.

**Methods:** Serum samples were collected from 24 healthy volunteers before and at three time points after a 30-minute walking stress test performed on three test days. In each experimental session, one of three ambulatory loads was applied: 100% body weight (BW); 80%BW; 120%BW. Serum concentrations of COMP, MMP-3, MMP-9, ADAMTS-4, PRG-4, CPII, C2C and IL-6 were assessed using commercial enzyme-linked immunosorbent assays. A two-stage analytical approach was used to determine the suitability of a biomarker by testing the response to the stress test (criterion I) and the dose-response relationship between ambulatory load magnitude and biomarker kinetics (criterion II).

**Results**. COMP, MMP-3 and IL-6 at all three time points after, MMP-9 at 30 and 60 minutes after, and ADAMTS-4 and CPII at immediately after the stress test showed an average response to load or an inter-individual variation in response to load of up to 25% of pre-test levels. The relation to load magnitude on average or an inter-individual variation in this relationship was up to 8% from load level to load level. There was a positive correlation for the slopes of the change-load relationship between COMP and MMP-3, and a negative correlation for the slopes between COMP, MMP-3 and IL-6 with MMP-9, and COMP with IL6.

**Conclusions:** COMP, MMP-3, IL-6, MMP-9, and ADAMTS-4 warrant further investigation in the context of articular cartilage mechanosensitivity and its role in joint degeneration and OA. While COMP seems to be able to reflect a rapid response, MMP-3 seems to reflect a slightly longer lasting, but probably also more distinct response. MMP-3 showed also the strongest association with the magnitude of load.

## Introduction

Physical activity is a prerequisite for maintaining a healthy musculoskeletal system. For instance, physical activity in healthy adults reduces the risk of cartilage thinning, cartilage defects, and bone marrow lesions ^1^ although the protective role of joint loading in a physiologic biomechanical and biochemical environment is still controversial ^2^. Moreover, biomechanical risk factors for cartilage degeneration in conditions such as obesity, diabetes (metabolic syndrome), malalignment or trauma with joint injury ^3^ illustrate the central role that mechanical factors can have in an altered biomechanical and/or biochemical setting in osteoarthritis (OA) development and progression ^4^. Although physical activity can relieve OA symptoms ^5^, its role in maintaining healthy cartilage and in the initiation and progression of OA in humans remains largely unknown. Answering the central research question of how living articular cartilage responds to joint loads experienced during daily activities represents a major milestone towards understanding articular cartilage health and if a disruption of this response may play a role in the pathomechanics of OA. Here, we are particularly interested in the acute response to 30 minutes of ambulatory load in healthy individuals.

Generally, physiologic magnitudes of mechanical loading suppress the proinflammatory and catabolic effects of interleukin (IL)-1, while injurious magnitudes of loading activate proinflammatory and catabolic pathways leading to cartilage degradation ^6^. For instance, dynamic compression of articular cartilage explants at physiologic magnitudes blocks IL-1-induced increases in the mRNA levels of the degradative enzymes A disintegrin and metalloproteinase with thrombospondin motifs (ADAMTS)-4, ADAMTS-5, matrix metalloproteinase (MMP)-1, MMP-3 ^7^ and aggrecan break-down ^8^. In the same experimental set up, metalloproteinase inhibitor (TIMP)-3 expression increased. These results suggest a net decrease in MMP activity under these conditions ^7^. In ex vivo experiments, the expression of MMP-1, MMP-3, MMP-8 and MMP-13 significantly decreased in articular cartilage with loads below 0.5 N and the expression of MMP-1 and MMP-13 significantly increased with loads of 1 N ^9^. Moreover, load reduced MMP-1 and MMP-3 synthesis in situ in healthy but not in OA human cartilage suggesting that MMPs play a key role in regulating the balance of structural proteins of the articular cartilage matrix according to local mechanical demands ^10^. Similarly, excessive and continuous cyclic mechanical stress induced load-dependent production of MMP-9 in cultured chondrocytes ^11^. Similar observations have been later reported by Nakyama *et al.* ^12^. In vivo, increases in IL-6, tumor necrosis factor (TNF)-α, and MMP-9 were greater after a marathon than after a half-marathon ^13^.

Cartilage oligomeric matrix protein (COMP) is a prominent constituent of articular cartilage ^14^. Serum concentrations of COMP fragments are elevated in patients with knee OA ^15^ and decrease during immobilization in healthy persons ^16^ or joint injury ^17, 18^. Patients with greater serum COMP concentration experience a faster progression of the disease ^19^. It has been suggested that COMP molecules are important for maintaining the properties and integrity of the collagen network ^20^, contribute to the material properties of biological tissue ^21^, and transfer forces from the cartilage matrix to the cell ^22^. Thus, COMP may be an indicator for the relationship between mechanical loading of articular cartilage and biological or pathogenic processes. COMP is upregulated following cyclic compression in situ ^22^, and results of in vivo studies ^23^ in competing marathon runners suggested that mechanical variations in the way individuals perform the same activity are related to the differences in serum COMP levels.

Type II collagen is a major articular cartilage constituent, representing 90 to 95% of its total collagen content and forming the fibrils that give cartilage its tensile strength. In the process of collagen fibril formation—which is enhanced in OA cartilage ^24^—the C-propeptide is removed from the procollagen extracellularly and directly reflects the rate of type II procollagen synthesis (CPII) ^24^. Cleavage of type II collagen by collagenases is also excessive in OA cartilage ^25^. It yields fragments such as the COL2−3/4Clong mono epitope (C2C) ^26^ reflecting degradation. A smaller synthesis/degradation (CPII/C2C) ratio has been associated with an increased odds ratio for OA progression ^27, 28^ and this ratio increased after load-modifying joint distraction reflecting cartilage regeneration ^29^. Proteoglycan 4 (PRG-4) or lubricin is a proteoglycan that in humans is encoded by the PRG-4 gene and highly expressed by superficial zone chondrocytes and synoviocytes ^30^. PRG-4 is critical for maintaining appropriate boundary conditions of articulating joint surfaces (low friction). In anterior cruciate ligament transected joints in rats, joint exercise and hence load decreased PRG-4 cartilage expression, increased cartilage degeneration and reduced superficial zone chondrocyte viability ^31^. PRG-4 plasma levels in patients with advanced knee OA are lower than in healthy controls ^32^. In recent systematic reviews, COMP, MMP-1 and -3, C2C, and IL-6 were among the most promising prognostic biomarkers for knee OA ^33– 35^. Moreover, pre-operative ADAMTS-4 synovial fluid levels predicted outcome in patients with knee OA after high-tibial osteotomy, a load altering joint preserving surgical intervention ^36^. Serum ADAMTS-4 was elevated particularly in patients with early OA and genes associated with ADAMTS-4 participate in collagen metabolism ^37^. Hence, these blood markers are potential surrogates of biological processes in human articular cartilage and involved in OA pathophysiology.

**Figure 1.**
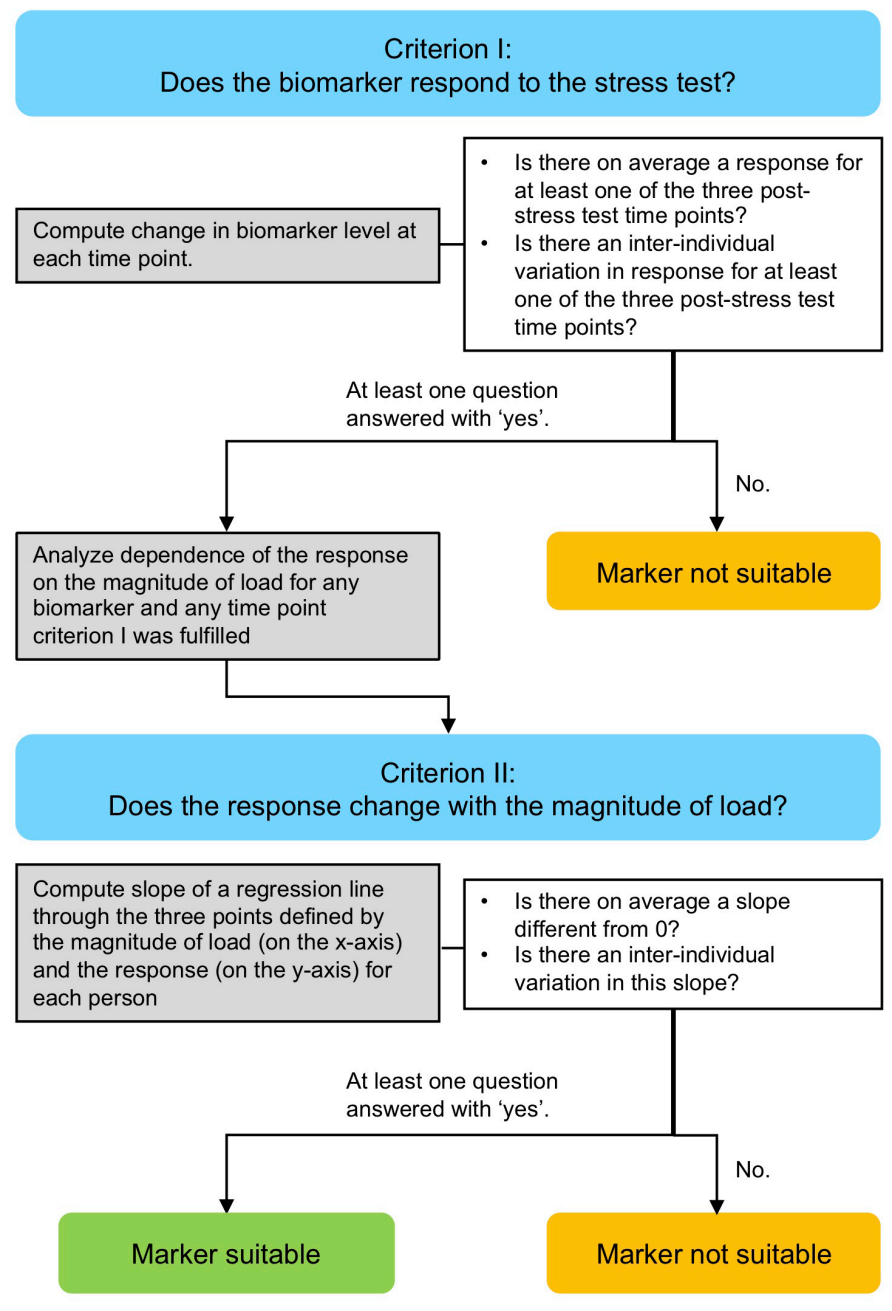
Flow diagram of the analytical approach. The approach comprised two stages consisting of a criterion (I and II, blue), statistical analysis of experimental data (grey) and criteria testing (white) for determining the suitability of each of nine potentially mechanosensitive markers for quantifying the dose-response relationship between ambulatory load magnitude and serum kinetics of mechanosensitive markers.

Joint load as experienced during ambulation translates to hydrostatic pressure and compressive, tensile, and shear forces in the extracellular matrix that are transduced to the chondrocyte ^38, 39^ resulting in altered turnover of matrix constituents via changes in signaling and regulation of catabolic and anabolic enzymes ^40, 41^. In vivo assessment of these interactions in human articular cartilage can only be made by measurements of surrogates of cartilage metabolism (blood markers and urine markers) and estimations of ambulatory load. Several studies ^16, 23, 42– 47^ have investigated the effects of ambulatory exercises (walking, cycling, running, marathon and ultramarathon) or immobilization on blood levels of candidate surrogates for cartilage metabolism. All previous studies reported biomarker kinetics during and up to at least 1 hour after the exercise bout. Based on the literature on exercises of varying intensity, one would expect that greater ambulatory load would result in greater biomarker response. However, to date the dose-response relationship between the magnitude of ambulatory load and serum kinetics of mechanosensitive blood markers in vivo in humans is unknown. The purpose of this study was to determine the suitability of selected blood biomarkers of articular cartilage as mechanosensitive markers and to investigate the dose-response relationship between ambulatory load magnitude and marker kinetics in response to load. We conducted an ambulatory loading experiment with repeated blood sampling with three different ambulatory load magnitudes and applied a two-stage analytical approach ( Figure 1) for determining the suitability of each of nine potentially mechanosensitive markers (COMP, MMP-3, MMP-9, ADAMTS-4, PRG-4, CPII, C2C, CPII/C2C and IL-6) for quantifying this dose-response relationship. We hypothesized that some but not all candidate markers would show a dose-response relationship and that the biomarker kinetics in response to load and the dose-response relationship would correlate among mechanosensitive markers.

## Methods

This study is an extension of our previously published study on the dose-response relationship between physiological stress and serum COMP levels in healthy subjects ^48^. We combined an experimental approach and an analytical approach as illustrated in Figure 1.

### Experimental approach

***Participants.*** Twenty-four healthy persons volunteered to participate in this study (12 female, mean ± standard deviation, age: 25.7 ± 1.4 years; body height: 1.67 ± 0.09 cm; body mass: 62.7 ± 8.4 kg; body mass index (BMI): 22.3 ± 1.6 kg/m ^2^; 12 male, age: 25.0 ± 2.2 years; body height: 1.81 ± 0.08 cm; body mass: 79.1 ± 11.6 kg; BMI: 24.0 ± 2.7 kg/m ^2^). Participants were recruited from July to September 2017 from the local community via advertisement on the institutional website until the desired numbers were reached. Only persons meeting the following inclusion criteria were considered: age between 18 and 30 years; physically active (>2 times/week); BMI below 30 kg/m ^2^; and no previous lower extremity injury and neuromuscular conditions that could have affected their gait. The study was approved by the regional ethics board (Ethikkomission Nordwestschweiz; EKNZ 2017-01006), registered at clinicaltrials.gov (NCT03455010) and conducted in accordance with the Declaration of Helsinki. Written informed consent was obtained from all participants prior to participation.

**Figure 2.**
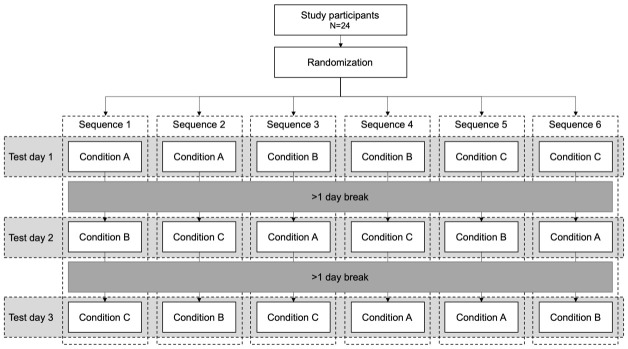
Flow chart of block randomized cross-over design.

***Experimental design.*** We used a block randomized crossover design as implemented in our previous study ^48^. Conditions were block randomized by the project manager in two blocks of all possible condition orders per sex to prevent a potential systematic condition effect ( Figure 2). Participants were enrolled by the project manager. Sealed envelopes were drawn from a container (one container per block) by the tester. Blinding to the experimental condition was not possible because of the obvious differences between conditions (partial weight bearing and additional load). However, the person processing the data was blinded to the condition. Because it does not seem feasible that a subject can actively alter the load-induced changes in blood markers of articular cartilage, it was assumed that this approach is appropriate for answering the research questions.

Participants completed a 30-minute walking stress test (hereafter termed ‘stress test’ on a treadmill (mercury® 3p, h/p/cosmos sports & medical GmbH, Nussdorf-Traunstein, Germany) on three different days at the Functional Biomechanics Laboratory, Department of Orthoapedics and Traumatology, University Hospital Basel, Switzerland. One of three ambulatory loads was applied per day: normal body weight (BW) (100% BW = normal load); reduced body weight (80% BW = reduced load); increased body weight (120% BW = increased load). An h/p/cosmos airwalk® system (h/p/cosmos sports & medical GmbH, Nussdorf-Traunstein, Germany) was used to dynamically unload the participant’s body during the unloading condition. The participant was placed in a harness connected to a pneumatic pulley system. The system was set to lower the participant’s BW by 20%. For the increased load condition, participants wore an adjustable weight vest (CAPITAL SPORTS Monstervest 20 kg, Chal-Tec GmbH, Berlin, Germany) with adjustable weights (1 kg increments) corresponding to 20% BW that were secured in pockets attached to the front and back of the vest. The amount of unloading and additional loading during the experiment was confirmed by measurements of the instrumented treadmill as previously described ^48, 49^.

For each participant, the schedule of test days was standardized, and only the load condition was modified. The different loading conditions were tested at the same time of day on three different days interspersed by at least one rest day. No sports or running and moderate to strenuous activities were allowed during the 24 h prior to each appointment. On each test day, participants fasted 1 h before and during the entire experiment. Five blood samples were obtained by venipuncture: 30 minutes before the stress test (t _-1_); immediately before the stress test (t _0_); immediately after the stress test (t _1_); 30 minutes after the stress test (t _2_) and 60 minutes after the stress test (t _3_).

Participants remained seated for 60 minutes before and for 60 minutes after the stress test. Each participant walked for 1 minute on the treadmill while the treadmill speed was continually increased to determine their individual preferred walking speed that was recorded and used for all stress tests for this participant. Then, the participant stood next to the treadmill while the weight vest or unloading harness was adjusted. During the stress test, participants walked on the treadmill for 30 minutes at their predetermined individual preferred speed. Immediately after the stress test, participants returned to their seated position.

***Blood samples.*** Venous blood samples were taken from the antecubital vein. A vein catheter (Vasofix® Safety PUR 20G, B. Braun Melsungen AG, Melsungen Germany) was placed during the rest period before the first blood sample at t _-1_ using sterile, disposable equipment and secured by tape. The catheter remained in the vein for 2.5 h. After every blood sample, the catheter was flushed with 10 ml isotonic saline solution (0.9% NaCl) to prevent plugging by clotting blood. The first 3 ml of each sample were discarded to avoid dilution through the injected saline solution. The blood samples clotted in the blood tubes (S-Monovette® 7.5ml Z-Gel, Sarstedt AG, Nürnbrecht, Germany) for 30 minutes. Subsequently, they were centrifuged (Sarstedt AG &Co SMC6) for 15 minutes at 2016 g, separated into aliquots and frozen (-20°C). The tubes were transferred to a -80°C freezer within 48 h until assayed.

Serum biomarker levels were measured using commercial enzyme-linked immunosorbent assays (Human COMP protein ELISA kit, BioVendor, Modrice, Czech Republic; Human Total MMP-3 and MMP-9 Immunoassays, R&D Systems Inc., Minneapolis, USA; Human Proteoglycan 4 (PRG-4) ELISA Kit, CUSABIO Technology LLC, Houston, USA; Human ADAMTS-4 ELISA Kit, Sigma-Aldrich, Saint Louis, USA; Human IL-6 Immunoassay, R&D Systems Inc., Minneapolis, USA; Human C2C and CPII ELISA kits, IBEX Pharmaceuticals Inc., Montréal, Canada). All samples were analyzed in duplicates. Intra-assay variability was estimated as coefficient of variation between the duplicates and were 2.2 ± 2.0% for COMP; 2.6 ± 1.7% for MMP-3; 1.2 ± 1.1% for MMP-9; 1.3 ± 1.3% for ADAMTS-4; 4.8 ± 3.8% for PRG-4; 2.4 ± 2.7% for IL-6; 8.1 ± 6.5% for C2C; and 3.0 ± 2.7% for CPII. The mean of the duplicates for each time point and condition was used for further analysis. The ratio between CPII and C2C was considered as an additional biomarker of synthesis/degradation ^26, 27^. The ratio was defined as the inverse of the ratio considered in the original publication because we expected a stimulation of tissue synthesis in a healthy population in response to a stress test (CPII:C2C – synthesis/degradation).

### Analytical approach

We defined a series of criteria to depict the suitability of a mechanosensitive biomarker ( Figure 1). Criterion I focused on the question of whether the biomarker responds to the stress test and was phrased as two questions:

Ia) Is there on average a response to the walking exercise for at least one of the three post-stress test time points?

Ib) Is there an inter-individual variation in the response to the walking exercise for at least one of the three post-stress test time points?

If at least one of these two questions was answered with ‘yes’, the dependence of the magnitude of the response on the magnitude of load was analyzed for any time point the criteria was fulfilled.

The dependence of the magnitude of the response of the biomarkers on the magnitude of the load was addressed at the individual level. Within each individual, we investigated at each time point how the response changes with the load by considering the slope of a regression line through the three points defined by the three ambulatory load levels (80% BW, 100%BW and 120%BW) (on the x-axis) and the magnitude of the response (on the y-axis). Consequently, Criterion II addressed the questions:

IIa) Is there on average a slope different from 0?

IIb) Is there an inter-individual variation in this slope?

If at least one of these two questions was answered with ‘yes’ for a specific biomarker at least one time point, then this biomarker was regarded as suitable for assessing the dose-response relationship between ambulatory load magnitude and biomarker kinetics in response to load. Note that question IIa answers also the question about a relation of the response to the magnitude of load at the population level because an average slope of 0 is equivalent to no trend in the load level specific mean response values at the population level.

If we can establish the suitability of several biomarkers using this approach, the natural question arises whether each biomarker can provide independent information or whether the different biomarkers measure the same. We hence also investigated the correlation of the individual changes and the individual slopes, respectively, between the different biomarkers.

### Statistical analysis and sample size estimation

We investigated the impact of the stress test and the load on the biomarker kinetics by considering the change from the baseline measurements at t _0_. Both the change in the absolute and the relative biomarker concentrations were considered, as it could not be decided *a priori* which type of concentration is more relevant. We used definitions trying to minimize potential drawbacks of absolute respective relative concentrations. For the absolute changes we used


$$\frac{y_{t}\;-\;y_{0}}{m}*\;100\;(1)$$

where *y _t_* denotes the concentration at the time points t _1_, t _2_, and t _3_, *y _0_* the concentration at t _0_, and *m* denotes the median value of the raw measurements at baseline (t _0_) (over all load levels and subjects) for the marker. Therefore, we can interpret the change in relation to the median value, and, for instance, a five-point change means that we observe a change corresponding to 5% of the median value. This way the absolute changes become comparable across the markers.

For the relative changes we shifted all measurements by a constant amount *c*, i.e. we used the formula


$$\frac{\left(y_{t}+c)-(y_{0}+c\right)}{y_{0}+c}\;+\;c\;*\;100\;=\;\frac{y_{t}-y_{0}}{y_{0}+c}\;*\;100\;(2)$$

The constant *c* is chosen such that the maximal relative change at the time points t _1_ and t _2_ overall load levels is limited to 75%. This way we diminish the instability of relative changes when *y*_0_ approaches 0.

To assess the investigated the impact of the stress test and the load on the biomarker concentration at a specific timepoint t > 0, the change measurements *δ _il_* for the individual *i* at load level *l* (with *l* coded as -1, 0 and 1) are modeled as


$$\delta_{il}\;=\;\alpha_{i}\;+\;\beta_{i}\;\times \;l\;+\varepsilon_{il}\;(3)$$

such that *α _i_* reflects the response to the stress test (at load level 100%) of individual *i* and *β _i_* the dependence on the load for individual *i*. These individual models were then combined into an overall mixed model assuming that the intercepts α _*i*_ and the slopes *β _i_* are drawn from a bivariate normal distribution. The mean *μ _α_* and the standard deviation σ _α_ of the intercepts reflect the average response to the walking exercise and the inter-individual variation in this response. The mean *μ _β_* and the standard deviation *σ _β_* of the slopes reflect the average dependence of the response on the load and the inter-individual variation in this dependence. Fitting such a mixed model provides us with estimates for these four quantities. In fitting the models, we used the restricted maximum likelihood techniques. P-values for the standard deviation were based on a likelihood ratio test using a mixture of two *χ* ^2^ distributions as reference, as suggested by Self and Liang ^50^.

A choice of *μ _α_* = *σ _α_* = *μ _β_* = *σ _β_* = 5 would reflect a situation of clinical interest. Based on 1000 simulations from the model we can conclude that we have a power of 98%, 87%, 98%, and 78%, respectively, to detect a difference from 0 at the 5% for each of the four parameters, if the standard deviation of the error term is 5. In the case of a standard deviation of 7, the power reduces to 96%, 54%, 93%, and 44%. Hence the chosen sample size of 24 is sufficient to declare markers of clinical interest as suitable with respect the average response or the average load, and also sufficient to detect inter-individual variation if the error variation is not too high.

With respect to the correlation between the biomarkers identified as suitable, we first considered the correlation in the individual changes. We try here to summarize the information from all three load levels by computing first the Spearman correlation at each load level and then taking the average. Inference for these average correlations was based on using the bootstrap and a normal approximation after applying Fisher’s z-transformation. In a second step we considered the correlation between the slopes. Here we computed the slope within each individual and reported the Spearman correlation.

All statistical computations were performed with Stata 15.1 (StataCorp LLC, College Station, TX, USA). All computations can also be performed in R (The R Foundation, Vienna, Austria). The level for statistical significance was set *a priori* to 5%.

### Ethics approval

This study was approved by the regional ethics committee (Ethikkommission Nordwestschweiz EKNZ 2017-01006).

### Participant consent

Written informed consent for participation and for publication of the participants details was obtained from all participants.

## Results

### Raw values

Raw measurements can be inspected at 51. Many parameters showed a substantial variation in resting levels between subjects. For ADAMTS-4, high intra-individual variation required the use of a logarithmic scale to visualize the data. In subject 110, the values of ADAMTS-4 fluctuated much more than in all other participants and were outside the detection limits, and hence we excluded this subject from the analysis of ADAMTS-4. For COMP, MMP-3 and IL-6, we observed a clear pattern in the response to the walking stress test over time, which was rather uniform across load levels and individuals. While for MMP-9, PRG-4 and ADAMTS-4, there were still similarities in the patterns across load levels at least for some individuals, for C2C, CPII and the CPII to C2C ratio, there appeared to be no common patterns.

Raw values were transformed to change values as described in the Methods sections. However, for ADAMTS-4, the absolute changes were based on log-transformed values (log10(1+x)) instead of the raw values.

### Criterion I: Does the biomarker respond to the stress test?

**Figure 3.**
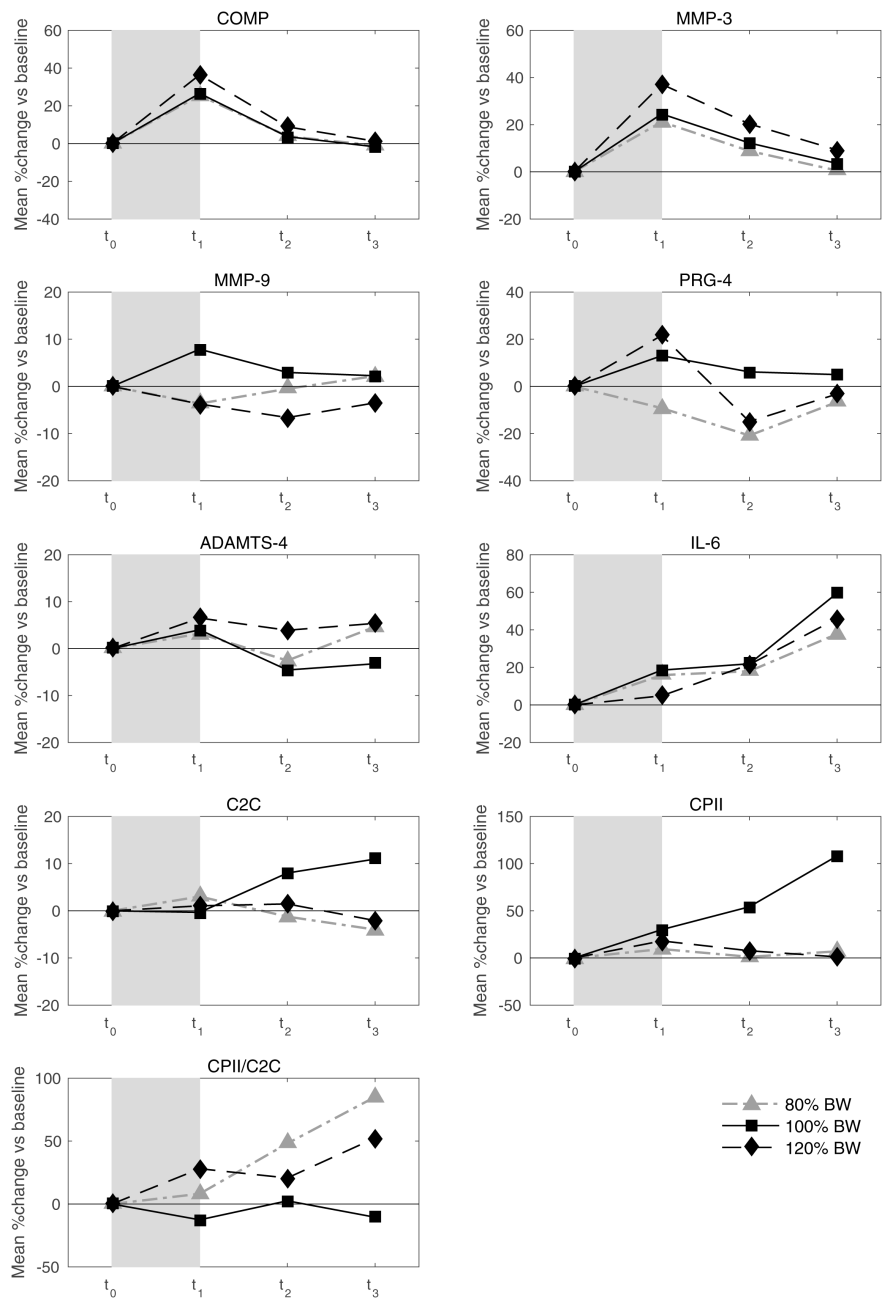
Mean trajectories in relative change describing biomarker kinetics in response to the walking exercise at each ambulatory load level for all biomarkers from immediately before (t _0_) to 60 minutes after (t _3_) a 30-minute walking exercise (grey area); COMP—cartilage oligomeric matrix protein; MMP—matrix metalloproteinase; PRG—proteoglycan; ADAMTS—A disintegrin and metalloproteinase with thrombospondin motifs; IL—interleukin; C2C—COL2−3/4Clong mono epitope; CPII—rate of type II procollagen synthesis.

**Figure 4.**
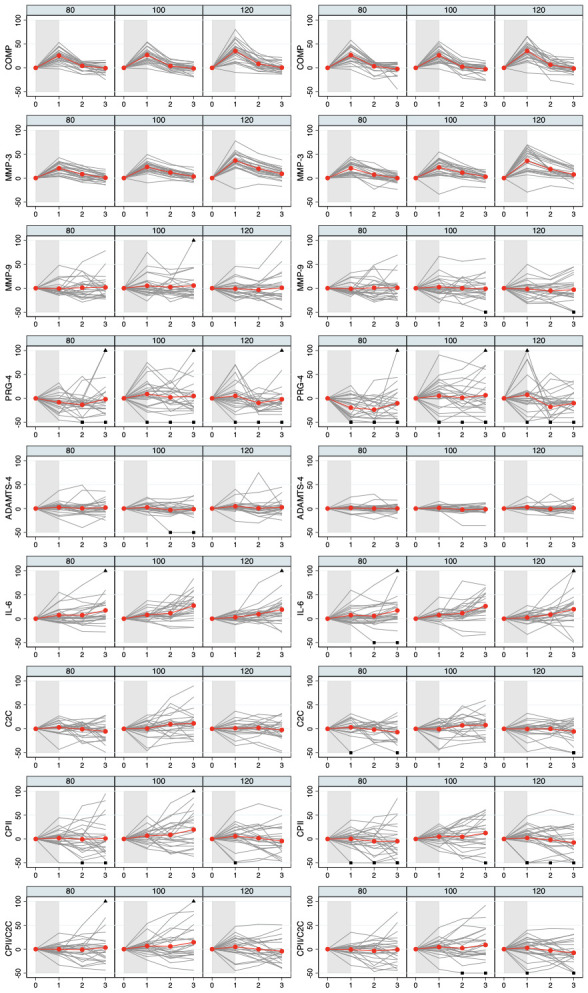
Individual and mean trajectories in relative change (left side) and absolute change (right side) describing biomarker kinetics in response to the stress test stratified by ambulatory load for all biomarkers from immediately before (t _0_) to 60 minutes after (t _3_) a 30-minute walking exercise (grey area); mean values of the trajectories are shown in red. Values above 100 and below 50 are truncated and marked with a triangle or a square, respectively. COMP—cartilage oligomeric matrix protein; MMP—matrix metalloproteinase; PRG—proteoglycan; ADAMTS—A disintegrin and metalloproteinase with thrombospondin motifs; IL—interleukin; C2C—COL2−3/4Clong mono epitope; CPII—rate of type II procollagen synthesis.

***Average response and inter-individual variation in response to walking exercise.*** Mean values of relative load-induced changes at all loads and all time points are shown in Figure 3. Individual and mean trajectories of the relative load-induced change of each biomarker are shown in Figure 4. Table 1 summarizes the evidence from this data on average response and inter-individual response variation. We observed a distinct average response to the stress test for COMP at t _1_ and t _2_ and for MMP-3 and IL-6 at all three time points after the stress test. For both COMP and MMP-3, we observed an average relative increase above 25% at t _1_. For COMP we observed a subsequent rapid decline to an average of about 5% at t _2_ and close to 0% at t _3_. For MMP-3, the decline was slower with an average of 13% at t _2_ and 4% at t _3_. For IL-6 we observed an increasing trend from an average increase relative to baseline of 6% at t _1_ to 9% at t _2_ to 21% at t _3_. There was some evidence for an average response for CPII and ADAMTS-4 at t _1_, but with rather low levels of less than 5% relative change. There was a significant average response for PRG-4 at t _2_, but because the average change was negative and not in agreement with the averages at the other two time points, we regarded this as a spurious finding. There was rather clear evidence for inter-individual response variation in COMP and MMP-3 at all time points and for IL-6 at t _2_ and t _3_. For COMP and MMP-3 the magnitude of the variation was similar with standard deviations of about 10% at t _1_ decreasing over time to about 5% at t _3_. For IL-6 the variation increased over time from about 5% at t _1_ to about 20% at t _3_. Interestingly, we also observed an inter-individual response variation for MMM-9 at time points t _2_ and t _3_, although there was no evidence for any average response. Considering absolute changes instead of relative changes lead to similar results ( Table 2), except that the inter-individual variation for MMP-9 lacked significance and PRG-4 showed a negative response at all time points.

**Table 1.** Relative changes: average response and inter-individual variation in response to the stress test.

*Biomarker*	*Time point*	*Mean*			*SD*		
		*Estimate*	*95% CI*	*P-value*	*Estimate*	*95% CI*	*P-value*
COMP	1	29.3	[23.8;34.8]	<.001	12.3	[8.7;17.5]	<.001
	2	5.5	[2.7;8.4]	<.001	5.9	[3.8;9.0]	<.001
	3	-0.4	[-3.1;2.3]	.783	4.9	[2.8;8.5]	.023
MMP-3	1	26.7	[22.2;31.2]	<.001	9.5	[6.4;14.1]	<.001
	2	13.1	[9.8;16.5]	<.001	6.9	[4.5;10.6]	.001
	3	4.2	[1.6;6.8]	.002	5.0	[3.0;8.3]	.010
MMP-9	1	1.1	[-3.1;5.3]	.597	0.0	[0.0;0.2]	1.000
	2	-0.1	[-5.1;5.0]	.977	9.0	[5.0;16.2]	.039
	3	2.7	[-5.7;11.1]	.525	16.3	[10.1;26.4]	.006
PRG-4	1	2.1	[-4.6;8.7]	.544	6.2	[0.6;65.7]	.666
	2	-6.9	[-12.8;∞]	.023	0.0	[0.0;∞]	1.000
	3	0.5	[-8.9;9.9]	.919	0.0	[0.0;∞]	1.000
ADAMTS-4	1	3.2	[0.7;5.8]	.014	0.0	[0.0; ∞]	1.000
	2	-0.7	[-4.8;3.3]	.722	0.0	[0.0; ∞]	1.000
	3	1.0	[-2.4;4.5]	.563	0.0	[0.0;∞]	1.000
IL-6	1	6.1	[3.0;9.2]	<.001	4.6	[1.9;11.3]	.219
	2	9.5	[4.0;15.0]	<.001	10.9	[6.9;17.4]	.003
	3	21.3	[12.3;30.2]	<.001	18.7	[12.3;28.4]	<.001
C2C	1	1.6	[-2.2;5.5]	.406	4.7	[1.2;18.5]	.446
	2	3.6	[-0.5;7.7]	.086	0.0	[0.0;∞]	1.000
	3	1.3	[-3.9;6.5]	.617	0.0	[0.0;∞]	1.000
CPII	1	4.9	[0.4;9.3]	.032	0.0	[0.0;∞]	1.000
	2	3.3	[-2.9;9.4]	.299	0.0	[0.0;∞]	1.000
	3	5.5	[-2.5;13.5]	.179	0.0	[0.0;∞]	1.000
CPII/C2C	1	3.7	[-0.1;7.6]	.058	0.0	[0.0;∞]	1.000
	2	1.5	[-3.9;6.9]	.583	0.0	[0.0;∞]	1.000
	3	4.4	[-3.0;11.9]	.239	0.0	[0.0;∞]	1.000

CI—confidence interval; SD—standard deviation; COMP—cartilage oligomeric matrix protein; MMP—matrix metalloproteinase; PRG—proteoglycan; ADAMTS—A disintegrin and metalloproteinase with thrombospondin motifs; IL—interleukin; C2C—COL2−3/4Clong mono epitope; CPII—rate of type II procollagen synthesis.

**Table 2.** Absolute changes: average response and inter-individual variation in response to the stress test.

*Biomarker*	*Time point*	*Mean*			*SD*		
		*Estimate*	*95% CI*	*P-value*	*Estimate*	*95% CI*	*P-value*
COMP	1	29.6	[24.1,35.1]	<.001	12.6	[9.0,17.7]	<.001
	2	4.2	[0.6,7.7]	.021	7.7	[5.3,11.2]	<.001
	3	-2.0	[-5.4,1.3]	.230	6.3	[3.8,10.5]	.011
MMP-3	1	26.2	[20.3,32.1]	<.001	12.5	[8.3,18.7]	<.001
	2	12.4	[8.2,16.6]	<.001	9.3	[6.5,13.4]	<.001
	3	3.3	[0.7,6.0]	.014	4.9	[2.9,8.5]	.019
MMP-9	1	-0.2	[-4.3,3.9]	.925	0.0	[0.0, ∞]	1.000
	2	-1.4	[-6.3,3.4]	.565	6.7	[2.3,19.2]	.307
	3	-1.1	[-7.4,5.2]	.739	8.7	[3.1,24.5]	.293
PRG-4	1	-2.3	[-10.6,6.0]	.594	0.0	[0.0, ∞]	1.000
	2	-13.3	[-22.0,inf]	.003	0.0	[0.0, ∞]	1.000
	3	-4.6	[-15.2,6.0]	.397	0.0	[0.0, ∞]	1.000
ADAMTS-4	1	1.8	[0.3,3.3]	.020	0.0	[0.0, ∞]	1.000
	2	-1.2	[-3.4,1.1]	.306	0.0	[0.0, ∞]	1.000
	3	-0.2	[-2.2,1.8]	.836	0.0	[0.0, ∞]	1.000
IL-6	1	5.8	[2.2,9.4]	.002	5.8	[2.8,12.0]	.120
	2	8.6	[2.0,15.2]	.010	14.0	[9.5,20.9]	<.001
	3	21.0	[10.5,31.6]	<.001	22.9	[15.7,33.4]	<.001
C2C	1	0.7	[-3.3,4.7]	.740	4.3	[0.7,26.1]	.570
	2	1.8	[-2.1,5.7]	.367	0.0	[0.0, ∞]	1.000
	3	-1.6	[-6.6,3.5]	.543	0.0	[0.0, ∞]	1.000
CPII	1	2.5	[-2.4,7.4]	.314	0.0	[0.0, ∞]	1.000
	2	-0.7	[-6.7,5.4]	.829	0.0	[0.0, ∞]	1.000
	3	0.2	[-7.2,7.6]	.964	0.0	[0.0, ∞]	1.000
CPII/C2C	1	2.1	[-1.8,6.0]	.286	0.0	[0.0, ∞]	1.000
	2	-1.4	[-6.8,3.9]	.602	0.0	[0.0, ∞]	1.000
	3	0.1	[-6.8,6.9]	.982	0.0	[0.0, ∞]	1.000

CI—confidence interval; SD—standard deviation; COMP—cartilage oligomeric matrix protein; MMP—matrix metalloproteinase; PRG—proteoglycan; ADAMTS—A disintegrin and metalloproteinase with thrombospondin motifs; IL—interleukin; C2C—COL2−3/4Clong mono epitope; CPII—rate of type II procollagen synthesis.

In summary, taking into account the criteria Ia and Ib stated in the Methods section, COMP, MMP-3 and IL-6 at all three post stress time points, MMP-9 at time point t _2_ and t _3_, and ADAMTS-4 and CPII at t _1_ were generally sensitive to the stress test and considered suitable mechanosensitive markers according to criterion I. All further investigations were hence restricted to these marker/time point combinations.

### Criterion II: Does the response change with the magnitude of load?

**Figure 5.**
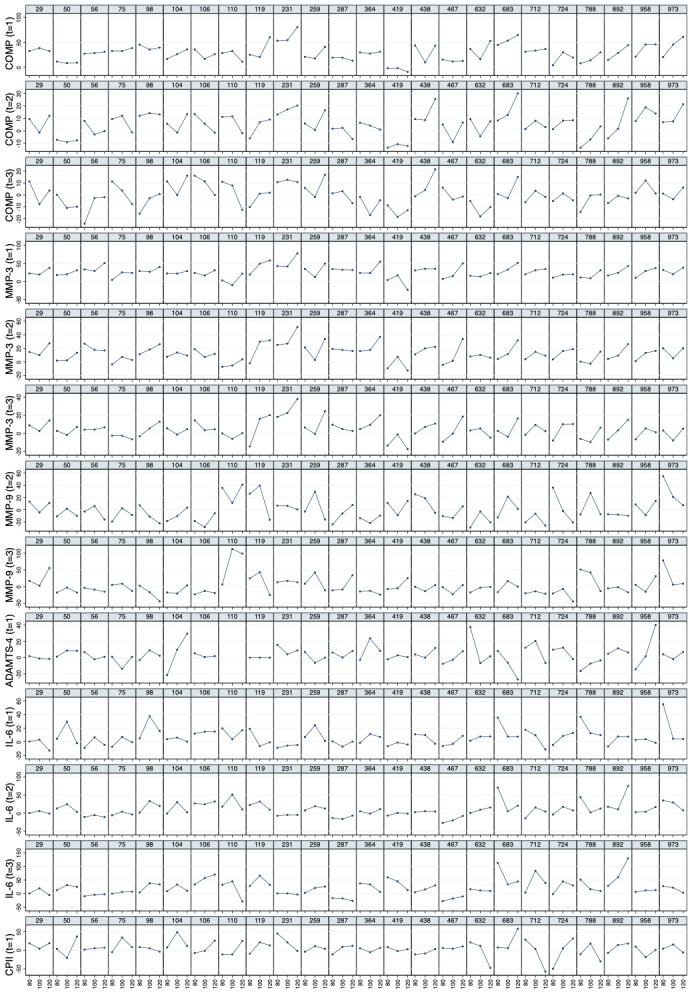
Relative change in biomarker concentration versus the load level for each individual for selected biomarkers and time points. COMP—cartilage oligomeric matrix protein; MMP—matrix metalloproteinase; ADAMTS—A disintegrin and metalloproteinase with thrombospondin motifs; IL—interleukin; CPII—rate of type II procollagen synthesis.

**Figure 6.**
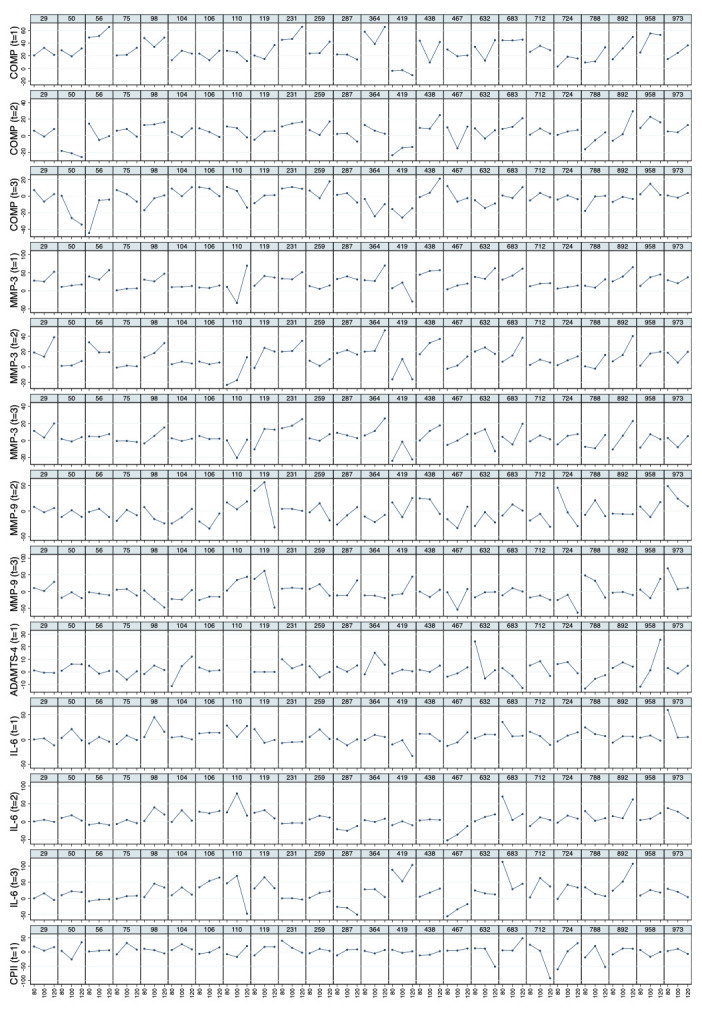
Absolute change in biomarker concentration versus the load level for each individual for selected biomarkers and time points. COMP—cartilage oligomeric matrix protein; MMP—matrix metalloproteinase; ADAMTS—A disintegrin and metalloproteinase with thrombospondin motifs; IL—interleukin; CPII—rate of type II procollagen synthesis.

***Average slopes and inter-individual variation in slopes describing the dose-response relationship.*** When considering the relation between the magnitude of load and the response for the selected parameter/time point combination in Table 3, we observed for MMP-3 an average slope clearly above zero for all time points. The relative change in MMP-3 increased by 8% from load level to load level (low – medium – high) at t _1_, and by 6% and 4% at t _2_ and t _3_, respectively. The relative change in COMP increased by five percentage points from load level to load level (low – medium – high) at t _1_ without a distinct increase at later time points. For all other parameters, there was little evidence for an average slope different from zero with estimates of the increase of less than 2.5 percentage points in absolute values. There was, however, some evidence for inter-individual variation in the slopes. This did not only hold for COMP and MMP-3 with standard deviations in the magnitude of three to four percentage points reaching significance at some time points but also for MMP-9, ADAMTS-4 and CPII with estimated standard deviations between five and 15 percentage points reaching also partially statistical significance. These insights were also corroborated when inspecting the individual data included in these computations ( Figure 5), indicating the existence of participants with nearly no change in response over the different load levels, participants with an increasing response from lowest to highest load, and participants with a decreasing response. The results with respect to the inter-individual variation were less clear for IL-6 with possible variation at t _1_. When considering the absolute changes ( Table 4 and Figure 6), we observed very similar results.

**Table 3.** Slopes of the relative change versus the magnitude of load: estimates of the mean and the standard deviation of the individual slopes.

*Biomarker*	*Time point*	*Mean*			*SD*		
		*Estimate*	*95% CI*	*P-value*	*Estimate*	*95% CI*	*P-value*
COMP	1	4.9	[1.7;8.1]	.003	4.0	[1.7;9.1]	.027
	2	2.1	[-0.2;4.4]	.074	3.9	[1.9;8.1]	.075
	3	0.6	[-1.9;3.1]	.645	3.7	[1.4;9.7]	.274
MMP-3	1	8.0	[4.9;11.2]	<.001	4.1	[1.8;8.9]	.022
	2	5.7	[3.2;8.2]	<.001	2.4	[0.2;31.9]	.161
	3	4.2	[1.9;6.5]	<.001	3.3	[1.2;9.5]	.041
MMP-9	2	-2.4	[-7.1;2.4]	.329	6.1	[2.6;14.3]	.042
	3	-0.5	[-7.7;6.7]	.894	10.7	[4.1;28.2]	.122
ADAMTS-4	1	1.0	[-3.2;5.2]	.630	8.4	[5.3;13.4]	.008
IL-6	1	-2.3	[-5.7;1.1]	.189	4.9	[2.3;10.7]	.033
	2	1.1	[-3.1;5.3]	.607	2.7	[0.4;17.9]	.296
	3	0.8	[-6.5;8.1]	.826	12.6	[6.4;24.8]	.108
CPII	1	2.0	[-5.2;9.2]	.582	14.0	[8.6;22.8]	.010

CI—confidence interval; SD—standard deviation; COMP—cartilage oligomeric matrix protein; MMP—matrix metalloproteinase; ADAMTS—A disintegrin and metalloproteinase with thrombospondin motifs; IL—interleukin; CPII—rate of type II procollagen synthesis.

**Table 4.** Slopes of the absolute change vs the magnitude of load: estimates of the mean and the standard deviation of the individual slopes.

*Biomarker*	*Time point*	*Mean*			*SD*		
		*Estimate*	*95% CI*	*P-value*	*Estimate*	*95% CI*	*P-value*
COMP	1	4.2	[1.4,7.1]	.004	2.1	[0.5,8.9]	.196
	2	1.6	[-0.7,4.0]	.175	3.4	[1.2,9.7]	.222
	3	0.4	[-2.7,3.5]	.804	5.5	[2.9,10.4]	.118
MMP-3	1	7.9	[3.6,12.1]	<.001	4.7	[1.8,12.2]	.060
	2	5.8	[3.2,8.4]	<.001	3.2	[0.8,13.4]	.287
	3	3.8	[1.5,6.0]	.001	2.2	[0.7,7.4]	.144
MMP-9	2	-3.0	[-8.6,2.7]	.301	9.2	[4.9,17.3]	.009
	3	-2.0	[-9.1,5.1]	.584	10.0	[3.3,30.0]	.319
ADAMTS-4	1	0.7	[-1.7,3.1]	.567	4.8	[2.9,7.8]	.020
IL-6	1	-2.5	[-6.0,1.0]	.159	3.0	[0.7,14.0]	.237
	2	1.5	[-3.0,5.9]	.519	4.2	[1.3,13.2]	.116
	3	1.3	[-5.9,8.5]	.723	10.6	[4.0,28.4]	.270
CPII	1	0.9	[-7.0,8.8]	.820	15.8	[10.0,25.1]	.003

CI—confidence interval; SD—standard deviation; COMP—cartilage oligomeric matrix protein; MMP—matrix metalloproteinase; ADAMTS—A disintegrin and metalloproteinase with thrombospondin motifs; IL—interleukin; CPII—rate of type II procollagen synthesis.

**Figure 7.**
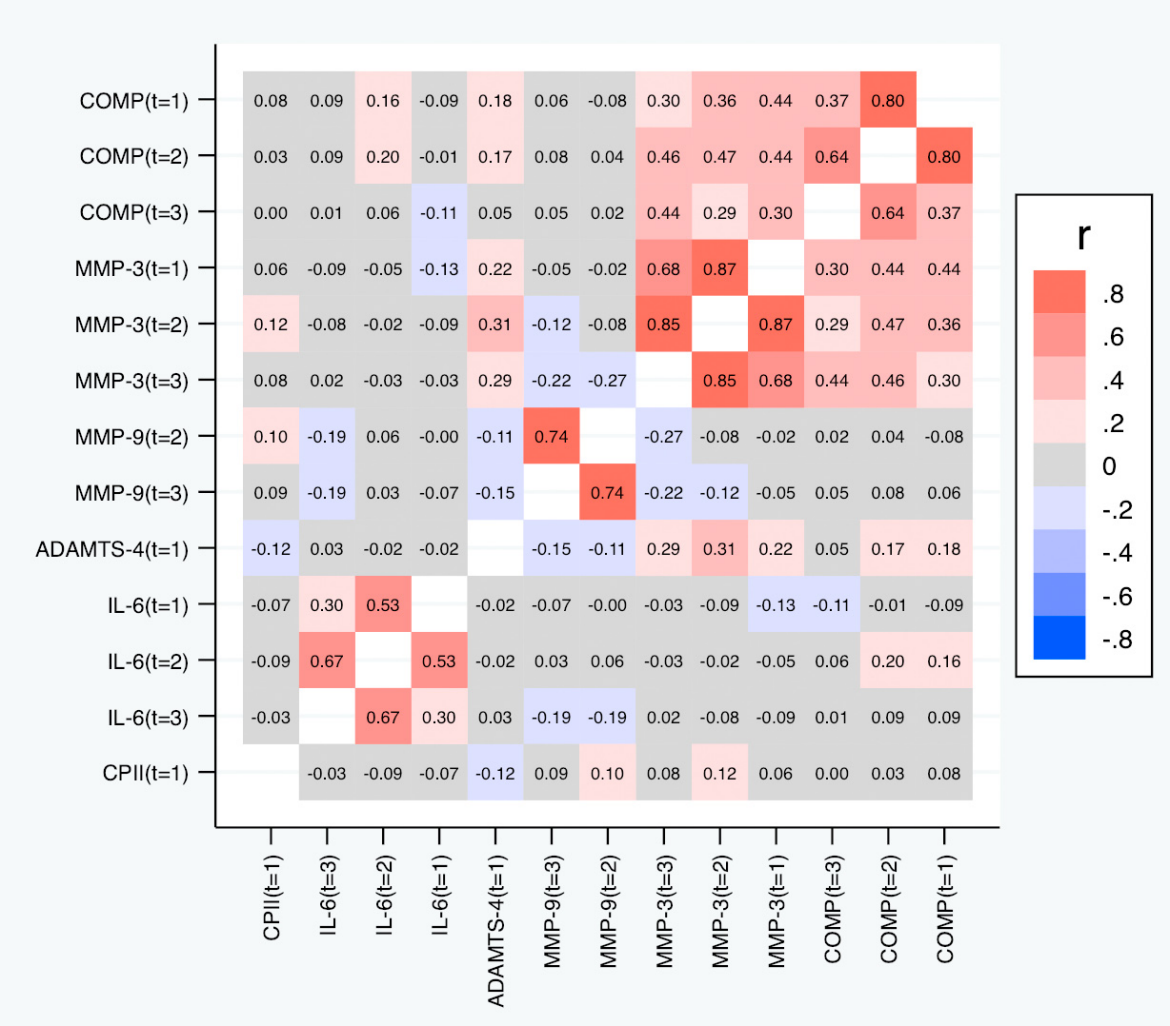
Heat map of the pairwise correlations of the relative change between the selected parameter/time point constellations. Shown are the estimated Spearman correlations (averaged over the three load levels). COMP—cartilage oligomeric matrix protein; MMP—matrix metalloproteinase; ADAMTS—A disintegrin and metalloproteinase with thrombospondin motifs; IL—interleukin; CPII—rate of type II procollagen synthesis.

**Figure 8.**
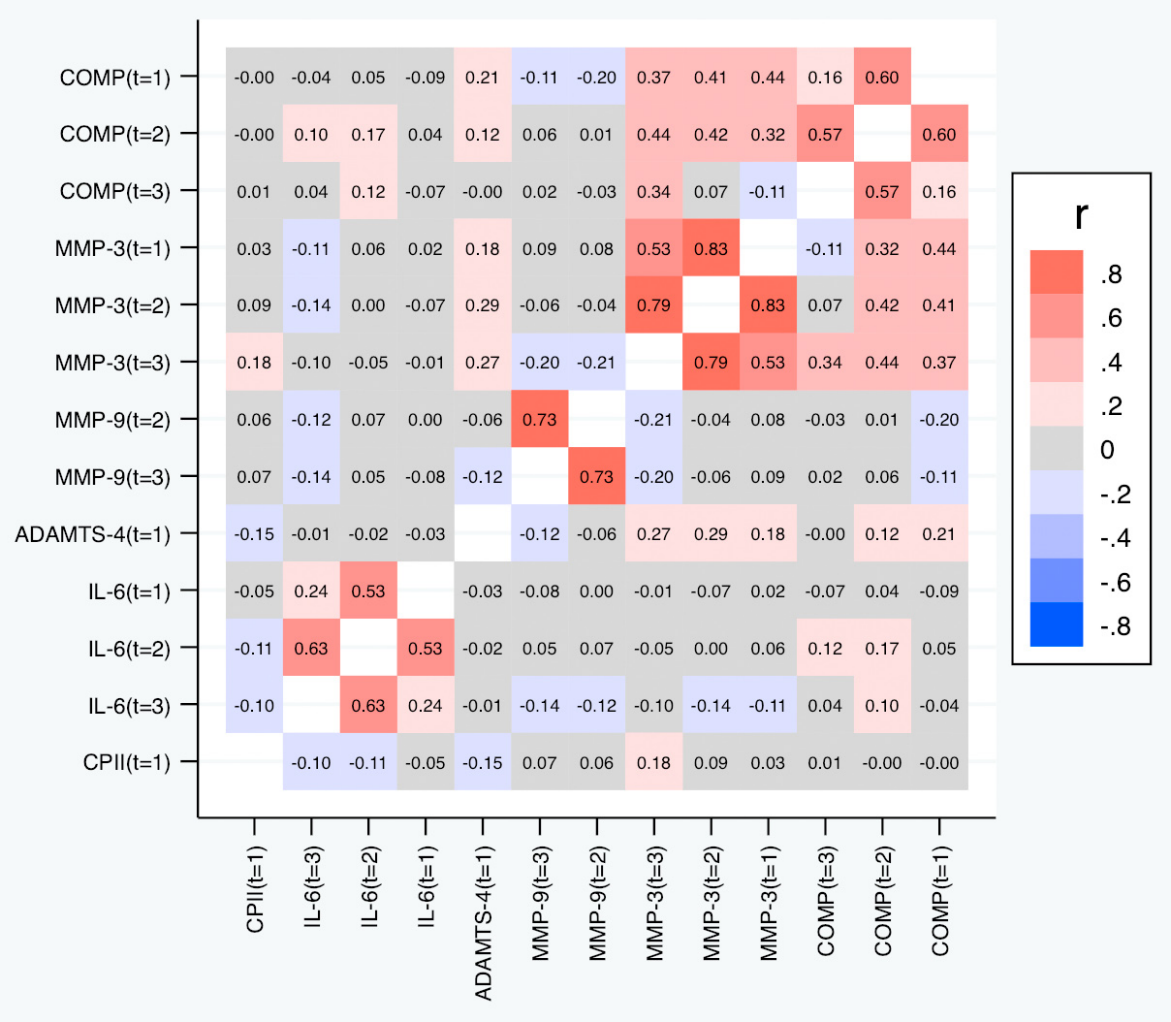
Heat map of the pairwise correlations of the absolute change between the selected parameter/time point constellations. Shown are the estimated Spearman correlations (averaged over the three load levels). COMP—cartilage oligomeric matrix protein; MMP—matrix metalloproteinase; ADAMTS—A disintegrin and metalloproteinase with thrombospondin motifs; IL—interleukin; CPII—rate of type II procollagen synthesis.

***Correlations among relative change variables.*** Table 5 and Figure 7 depict the correlations among the relative change variables that satisfied the criteria for a suitable marker. As expected, we observed substantial correlations among the change variables for the same marker at different time points except for the change in IL-6 at t _1_ and t _3_. The cross-marker correlations were generally rather low (|R| < 0.2) with the exception of the correlations between COMP and MMP-3 at the same time point but also across time points, and correlations between ADAMTS-4 at t _1_ and MMP-3 at all time points. A similar pattern was observed for the absolute change ( Figure 8).

**Table 5.** P-values of pairwise Spearman correlations of the absolute change between the selected parameter/time point constellations. Correlations with p-values below 0.05 are shown in bold.

	COMP (t=1)	COMP (t=2)	COMP (t=3)	MMP- 3 (t=1)	MMP- 3 (t=2)	MMP-3 (t=3)	MMP-9 (t=2)	MMP-9 (t=3)	ADAMTS-4 (t=1)	IL-6 (t=1)	IL-6 (t=2)	IL-6 (t=3)	CPII (t=1)
COMP (t=1)													
COMP (t=2)	***<.001***												
COMP (t=3)	***.021***	***<.001***											
MMP-3 (t=1)	***.002***	***<.001***	***.020***										
MMP-3 (t=2)	***.011***	***<.001***	***.042***	***<.001***									
MMP-3 (t=3)	***.053***	***.001***	***<.001***	***<.001***	***<.001***								
MMP-9 (t=2)	*.480*	*.720*	*.873*	*.877*	*.599*	***.047***							
MMP-9 (t=3)	*.645*	*.570*	*.721*	*.746*	*.457*	*.138*	***<.001***						
ADAMTS-4 (t=1)	*.188*	*.211*	*.694*	*.102*	***.010***	***.025***	*.386*	*.212*					
IL-6 (t=1)	*.645*	*.933*	*.465*	*.472*	*.629*	*.839*	*.996*	*.608*	*.899*				
IL-6 (t=2)	*.231*	*.144*	*.685*	*.757*	*.886*	*.851*	*.633*	*.862*	*.867*	***<.001***			
IL-6 (t=3)	*.610*	*.553*	*.914*	*.547*	*.554*	*.866*	*.076*	*.100*	*.810*	*.058*	***<.001***		
CPII (t=1)	*.503*	*.844*	*.976*	*.626*	*.284*	*.525*	*.423*	*.389*	*.377*	*.595*	*.490*	*.850*	

COMP—cartilage oligomeric matrix protein; MMP—matrix metalloproteinase; ADAMTS—A disintegrin and metalloproteinase with thrombospondin motifs; IL—interleukin; CPII—rate of type II procollagen synthesis.

**Figure 9.**
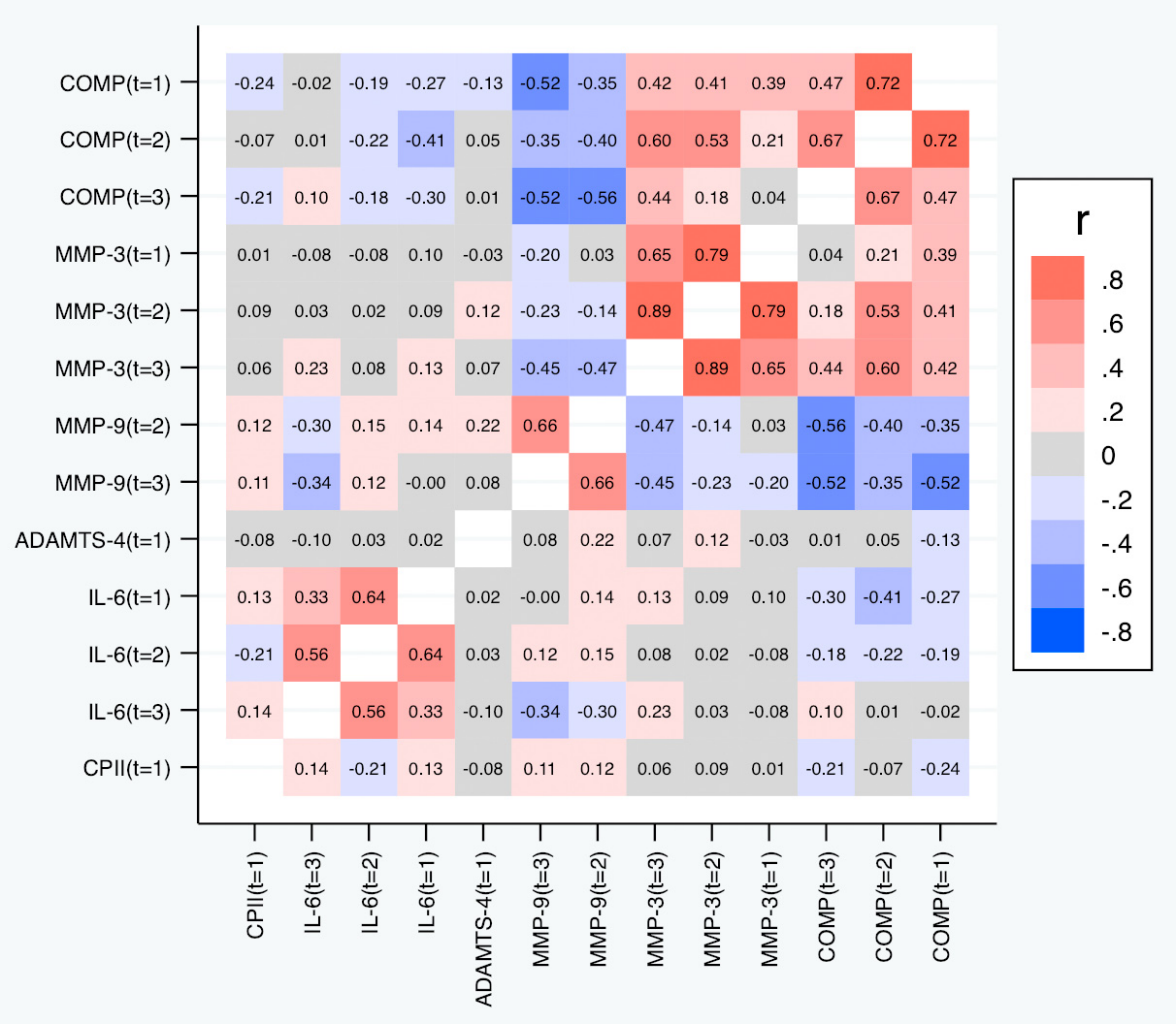
Heat map of the pairwise correlations of the slopes between the selected parameter/time point constellations based on the relative changes. Shown are the estimated Spearman correlations. COMP—cartilage oligomeric matrix protein; MMP—matrix metalloproteinase; ADAMTS—A disintegrin and metalloproteinase with thrombospondin motifs; IL—interleukin; CPII—rate of type II procollagen synthesis.

**Figure 10.**
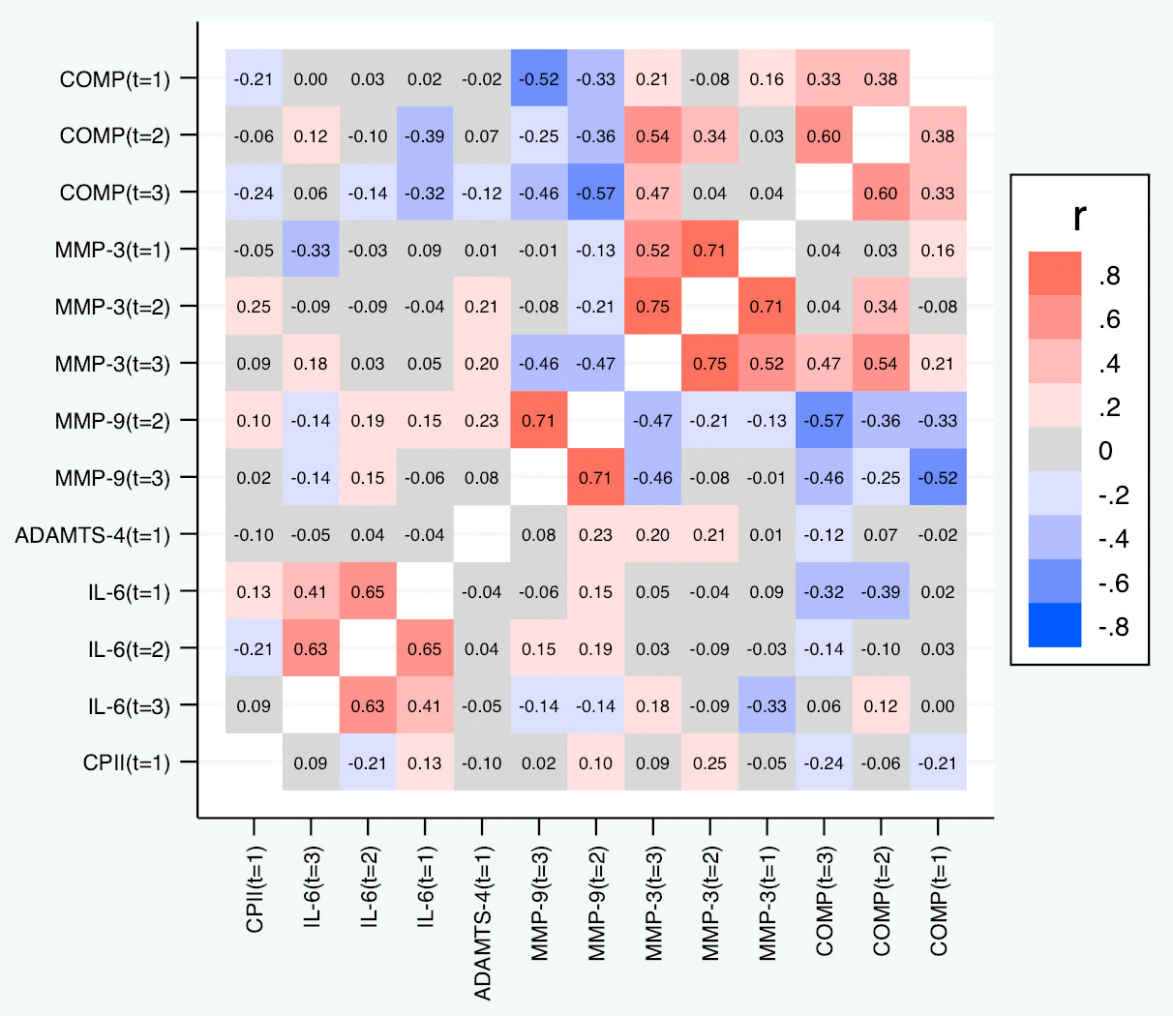
Heat map of the pairwise correlations of the slopes between the selected parameter/time point constellations based on absolute changes. Shown are the estimated Spearman correlations. COMP—cartilage oligomeric matrix protein; MMP—matrix metalloproteinase; ADAMTS—A disintegrin and metalloproteinase with thrombospondin motifs; IL—interleukin; CPII—rate of type II procollagen synthesis.

Table 6 and Figure 9 depict the correlations between the slopes based on the relative change. Again, we observed substantial correlations among the slopes for the same marker at different time points (contributing also to the conceptual validation of the slopes). Cross-marker correlations were again often low (|R| < 0.2), but we did not only observe some correlation between the slopes of COMP and MMP-3, but also a negative correlation between the slopes of COMP and MMP-9 (also across time points), and – to a lower degree – between the slopes of MMP-3 and MMP-9. Negative correlations were also observed between the slopes of IL-6 on one side and the slopes of COMP and MMP-9 on the other side at some time points. A similar pattern was observed for the slopes based on the absolute change ( Figure 10).

**Table 6.** P-values for pairwise Spearman correlations of the slopes between the selected parameter/time point constellations based on the relative changes. Correlations with p-values below 0.05 are shown in bold.

	COMP (t=1)	COMP (t=2)	COMP (t=3)	MMP- 3 (t=1)	MMP- 3 (t=2)	MMP- 3 (t=3)	MMP- 9 (t=2)	MMP- 9 (t=3)	ADAMTS-4 (t=1)	IL-6 (t=1)	IL-6 (t=2)	IL-6 (t=3)	CPII (t=1)
COMP (t=1)													
COMP (t=2)	**<.001**												
COMP (t=3)	**.062**	**.008**											
MMP-3 (t=1)	**.134**	.303	.861										
MMP-3 (t=2)	**.066**	**.008**	.398	**.002**									
MMP-3 (t=3)	**.071**	**.002**	**.027**	**<.001**	**<.001**								
MMP-9 (t=2)	**.086**	**.064**	**.006**	.916	.560	**.058**							
MMP-9 (t=3)	**.021**	**.093**	**.022**	.350	.252	**.018**	**<.001**						
ADAMTS-4 (t=1)	.499	.807	.960	.915	.619	.751	.361	.691					
IL-6 (t=1)	**.207**	**.077**	**.140**	.646	.711	.637	.584	.997	.929				
IL-6 (t=2)	.453	.373	.473	.726	.922	.707	.509	.629	.913	**.031**			
IL-6 (t=3)	.933	.965	.643	.665	.917	.298	**.177**	**.129**	.660	**.153**	**.005**		
CPII (t=1)	.297	.785	.373	.961	.674	.789	.577	.617	.753	.573	.373	.653	

COMP—cartilage oligomeric matrix protein; MMP—matrix metalloproteinase; ADAMTS—A disintegrin and metalloproteinase with thrombospondin motifs; IL—interleukin; CPII—rate of type II procollagen synthesis.

In summary, COMP, MMP-3, IL-6, MMP-9, and ADAMTS-4 fulfilled criterion II for assessing the dose-response relationship between ambulatory load magnitude and biomarker kinetics.

## Discussion

The aim of this study was to determine the suitability of selected blood biomarkers of articular cartilage as mechanosensitive markers and to investigate the dose-response relationship between ambulatory load magnitude and marker kinetics in response to load. We identified several promising markers. COMP seems to be able to reflect a rapid response to physiological stress, while MMP-3 seems to show a slightly longer lasting but also more distinct (greater and more consistent) response. MMP-3 showed also the strongest association with the magnitude of load. The metabolic response measured by IL-6 is also clearly long lasting, probably beyond the time span covered in our set up. Interestingly, this might also be the case for MMP-9, for which we could not observe a response on average, but nevertheless inter-individual variations of the changes and slopes at the late time points. For ADAMTS-4 and CPII, we also found signs of an inter-individual variation in the relation of the magnitude of load to the response at t _2_. These results strongly indicate that assessing one isolated biomarker in the context of loading (and unloading) experiments may not fully capture the metabolic dynamics in response to load. Moreover, it is possible that there are responders and non-responders to physiological stress or that the response is affected by factors such as disease or injury, which we did not investigate in this study.

The results of our experiment and analytical approach represents an extension of previous reports of changes in blood biomarker levels with load during physical activity, immobilization, and microgravity. For instance, previous studies ^23, 42– 45, 47^ have investigated the effects of walking, cycling, running, marathon and ultramarathon on blood levels of candidate surrogates for cartilage metabolism, most prominently on COMP. Walking for 30-minutes increased serum COMP levels in young healthy adults ^52^, in older healthy adults and in patients with knee OA ^53^. The dynamics of COMP after the exercise appeared to differ between these groups ^53^. Niehoff *et al.* ^43^ observed a 39% increase in COMP immediately after running for 30-minutes that remained elevated for up to 90 min after the running intervention before returning to the baseline level. In contrast, deep knee bends or lymphatic drainage did not elicit any changes in COMP levels. Recently, Bjerre-Bastos *et al*. ^47^ concluded that cyclic impact loading (running) elicits greater acute changes in biomarkers of cartilage extra-cellular matrix turnover than cyclic non-impact loading (cycling) as the mean change in COMP differed after running compared to after cycling but not after resting. Neidhart *et al.* ^42^ reported that during a marathon run, serum levels of IL-1RA, IL-6, TNF- *α* and COMP rose significantly, and gradually returned to baseline within 24 h. In another marathon study, differences in serum levels from before to after the run were observed for COMP, IL-6 and hsCRP but not for TNF-α ^45^. Mündermann *et al.* ^44^ found elevated COMP, MMP-9 and MMP-3 but not in MMP-1, C2C, CPII, and C2C:CPII throughout a multistage ultramarathon (4486 km). Interestingly, immobilization during bed rest studies has resulted in decreased levels of COMP, MMP-3, and MMP-9 but not MMP-1 and in elevated TNF-α levels ^16, 54^. Moreover, a delayed increase in COMP was observed in astronauts upon return to gravity after six months on the International Space Station ^55^. While some of these studies acknowledged the effect of different loads on blood biomarker dynamics, none of these studies quantified the accumulated load during the physiological stress or unloading condition. In this context, the question arises whether a greater response can be provoked with a higher than normal load and if individuals who are more sensitive to higher loads have subclinical cartilage and/or joint damage.

Remarkably, the mechanosensitive biomarkers identified in this study are biomarkers that are upregulated and mediate cartilage break-down in OA ^56, 57^. For instance, Georgiev *et al.* ^58^ have recently shown that serum COMP and MMP-3 levels were higher in patients with diagnosed knee OA and that patients with more severe knee OA had higher COMP levels than patients with less severe knee OA. These researchers also reported that MMP-3 levels were higher in the generalized OA compared to the isolated knee OA group and suggested that COMP may reflect knee structural damage while MMP-3 may reflect OA “generalization”. Moreover, our results of a correlation between COMP and MMP-3 levels among healthy subjects confirm previously reported ^58^ correlations between these biomarkers across healthy persons and patients with OA. Interestingly, five participants in our study had resting COMP levels above the cut-off value of 717.5 ng/mL (sensitivity 73.2% and specificity 71.0%) to differentiate between controls and patients with knee OA, and one participant after the walking exercise even reached COMP levels above the cut-off value of 1185 ng/mL for OA (100% specificity with 33.9% sensitivity) as reported by Georgiev *et al.* ^58^. In contrast, MMP-3 levels in all participants were well below the cut-off values to differentiate between controls and patients with knee OA ^58^. This observation suggests that the response in MMP-3 levels to ambulatory load may be more sensitive to exercise in healthy joints than that in COMP levels because none of our subjects had symptoms of OA and we would have not expected levels above the cut-off values for OA. COMP levels above cut-off values also emphasize the need for standardized testing protocols because of the mechanosensitivity of the markers and hence that loading can result in increased values in healthy individuals that are in the range of OA.

To the best of our knowledge, this is the first study to show changes in serum ADAMTS-4 depending on the load magnitude in vivo in humans. ADAMTS-4 is induced by IL-6 and cleaves COMP ^59^. High loading regimen (high stress, excessive duration) have been shown to increase ADAMTS-4, and aggrecan degradation in human articular cartilage explants is mediated by ADAMTS-4 ^60^. Moreover, genes associated with ADAMTS-4 participate in collagen metabolism ^37^. The results of our study suggest that load experienced during daily activities may be sufficient to influence metabolic pathways and transport of markers, and hence may play a role in maintaining healthy articular cartilage and potentially in degenerative processes in OA. Moreover, slight modifications in the load magnitude such as when wearing a backpack seem to have a profound effect on the response of the tissue to the exercise. In a mechanical loading experiment on articular cartilage explants, Schätti *et al.* ^61^ showed that sliding loads that increase extracellular matrix deformation/strain induce enzyme-mediated catabolic processes in articular cartilage explants reflected by increased ADAMTS-4 (and MMP-3) gene expression. Hence, the combination of modified load magnitude with situations of joint instability such as after rupture of the anterior cruciate ligament may be particularly precarious for initiating or accelerating degenerative processes leading to premature OA.

Interestingly, IL-6 responded to the physiological stress but did not show a dose-response relationship in our study, yet we observed correlations between IL-6 and COMP and MMP-3 after loading suggesting that IL-6 may play an important role in the context of mechanosensitivity of articular cartilage biomarkers. In general, IL-6 levels increase exponentially after strenuous exercise, and the magnitude of the increase depends on the length and intensity of the exercise ^62^. Even a walking exercise – although with walking distances beyond 30 km per day – may result in a 40-fold increase in IL-6 levels ^63^. While several studies have reported a peak in IL-6 levels at the end of an exercise ^64– 66^, in our experiments IL-6 increased exponentially after completing the exercise. IL-6 is produced locally in the skeletal muscle in response to exercise ^67^, and the IL-6 produced by contracting skeletal muscle may partly mediate exercise-related metabolic changes ^68^. Consequently, the lack of a distinct relationship between load magnitude and IL-6 levels suggest that the different loading conditions did not represent relevant differences in the demand on skeletal muscle further supporting our experimental framework of altering joint load without substantial changes in exercise intensity. We observed a large variability in increase in IL-6 in response to the walking exercise among subjects. This result is surprising because a 30-minute walking exercise at self-selected speed – whether with 20% lower or 20% greater body weight – in physically active healthy persons would be expected to be of similar intensity for all participants. However, in the current study we did not assess any parameters describing the associated cardiovascular stress such as heart rate or oxygen saturation, and hence this variability warrants further investigation.

In this study, we assessed the suitability of different biomarkers for assessing the dose-response relationship between ambulatory load and load-induced change in biomarker levels by looking at their increase relative to the pre-test values. Similarly, previous studies ^23, 42– 45^ have focused on assessing load-induced increases in candidate biomarkers. However, whenever we observe an initial increase, the response of the biomarker to the stress test is not only given by this increase but also by the subsequent decline, which may occur at a different rate. COMP and MMP-3 showed rather uniformly such an initial increase. We can observe in Figure 4 that this decline is again rather uniform and tends to compensate the initial increase. Hence, by analyzing this decline, we observed roughly the same results as when analyzing the initial increase (data not shown). It might be of interest to analyze also deviations from this general pattern to compensate the initial increase by looking at residuals from a corresponding prediction model, but this approach would require larger sample sizes. Therefore, assessing not only the variability in specific biomarker dynamics in response to a specific loading condition, but also the dependency of these dynamics to the load magnitude may be particularly useful for identifying persons who might be particularly sensitive to changes in load or at risk for articular cartilage degeneration.

Data obtained with an instrumented knee prosthesis revealed that compressive load at the knee can exceed twice the person’s BW during walking, stair walking, rising from a chair, squatting, and even during a golf swing ^69^, and peak loads during running can be as high as four times those experienced during walking ^70^. However, mechanical load is not only defined by its magnitude but also by the frequency of load application, time between load applications and number of loading cycles. Ambulation comprises different modes ranging from walking to running and sprinting for different durations (seconds to hours) and includes extreme exercises such as multistage ultramarathons. Interestingly, the load per unit distance travelled may be smaller in more demanding modes of ambulation (e.g., running: greater load magnitude and higher cadence but longer time between load application and fewer loading cycles per unit distance travelled) than in less demanding modes of ambulation (e.g. walking) ^70^. Hence, all characteristics of load must be considered when modulating the dose of ambulatory load, and ideally only one characteristics of load (e.g., load magnitude) is modulated while the other characteristics (e.g. cadence, duration) remain constant. As previously reported, we were able to modulate ambulatory load magnitude without relevant changes to loading frequency (cadence), duration, number of steps (constant walking speed across conditions), or joint kinematics in the current experimental setup ^49^.

The response of biomarkers to an exposure like a stress test is typically assessed by the change in the measured values from pre-stress test to post-stress test at the individual level. However, there are two ways to assess the change: we can consider the absolute change or the relative change compared to the pre-stress test measurements, and both approaches can be found in the literature ^16, 23, 42– 46^. Relative changes have the advantage that they take into account that changes are often roughly proportional to the pre-stress test value. They are also directly comparable across different markers, which is not the case for absolute changes. However, relative changes introduce additional variation and tend to be unstable if the pre-stress test values are close to zero. We investigated both approaches in this paper and used definitions trying to minimize the drawbacks and obtained very similar results. Consequently, our conclusions do not depend on the choice of considering absolute or relative changes.

Overall, we demonstrated that our experimental setup is well suited to study the dependence of the biomarker response to ambulatory activity on the magnitude of load and even the individual variation in the magnitude of load versus response relationship. The latter is in particular corroborated by correlations of the slopes across various markers. Using this experimental framework, we were able to modulate ambulatory load magnitude without any relevant changes in joint kinematics ^49^. Although participants were instructed to refrain from demanding physical activity during the 24 h prior to and from any exercise on the day of the respective experiments, we did not monitor their activity. However, the entire loading experiment (2.5 h) was controlled. As in all human experiments, we were limited to estimating metabolic changes in articular cartilage by assessing systemic changes in serum biomarker levels. It must be noted that commercial ELISA kits tools as used in the current study usually measure total protein and do not capture the small fractions of, for instance, MMP-3 proteolytically cleaved and activated as previously suggested in the disease process of rheumatoid arthritis ^71^. The current samples could be exploited in future analyses targeting epitopes and activity to offer further biological and mechanistic insight into the effect of well-defined mechanical stimuli on articular cartilage biology. Moreover, although we did measure the ground reaction force during all loading experiments confirming the desired loading or unloading ^49^, we were not able to directly measure ambulatory load at the tissue level. In this study, we did not collect imaging data on the study participants to confirm joint health but relied on their self-reported data concerning no injury history. Although at the age of younger than 28 years of age this is very unlikely, we cannot completely rule out the presence of early degenerative changes in all participants. Nonetheless, we believe that our current data set represents a major advance in the area of in vivo in human articular cartilage biomarker mechanosensitivity.

In this study, we chose to apply a targeted and conservative approach by assessing a set of specific serum markers that had been shown to be relevant for articular cartilage health and degeneration and/or responsive to acute loading using well-established methods (ELISAs). In a next step, the analyses could be expanded to targeted or untargeted metabolomic and proteomic approaches ^72, 73^ that may allow to discover novel markers with a possibly even stronger dose-response relationship ^74^. This framework forms the basis for elucidating whether the sensitivity of biomarker kinetics to different ambulatory loads changes with age or is disrupted by joint injury hence evidence of altered cartilage mechanosensitivity in the early stage of OA and for testing pharmacological interventions with using participants as their own controls.

## Conclusion

Our experimental framework and two-stage analytical approach appear well suited for studying the dependence of biomarker kinetics in response to ambulatory activity on the magnitude of load and even the individual variation in the magnitude of load versus response relationship in vivo in humans. We identified COMP, MMP-3, IL-6, MMP-9, and ADAMTS-4 as markers that warrant further investigation in the context of articular cartilage mechanosensitivity and its role in joint degeneration and OA. While COMP seems to be able to reflect a rapid response, MMP-3 seems to reflect a slightly longer lasting, but probably also more distinct response. MMP-3 showed also the strongest association with the magnitude of load. Interactions among selected marker kinetics in this experimental framework of altering joint load may represent an opportunity for investigating metabolic processes in response to load.

## Data availability statement

### Underlying data

Zenodo: Kinetics of selected blood biomarkers of articular cartilage in response to stress test with modulated ambulatory load. https://doi.org/10.5281/zenodo.4955982 ^51^

This project contains the following underlying data;

- raw data.pdf: Raw concentrations for each marker (COMP, MMP-3, MMP-9, ADAMTS-4, PRG-4, CPII, C2C, CPII/C2C and IL-6), participant, time point and condition.

It also contains the following reporting guidelines.

- trendstatement_TREND_Checklist_Mundermann.pdf: Completed TREND checklist

Data are available under the terms of the Creative Commons Attribution 4.0 International license (CC-BY 4.0).

## Authors’ contributions

AM and SH designed the study; SH recruited the participants, collected the data and processed the blood samples; SH, CN prepared the data for statistical analysis; WV performed the statistical analysis; all authors interpreted the data; AM, WV, AML and CE prepared the manuscript; all authors contributed to reviewing and revising the manuscript, and agreed on the final draft.
