# Supplementary material for: Experimental-analytical approach to assessing mechanosensitive cartilage blood marker kinetics in healthy adults: dose-response relationship and interrelationship of nine candidate markers
Source: F1000Res. 2022 Jan 11;10:490. Originally published 2021 Jun 22. [Version 2] doi: 10.12688/f1000research.52159.2 (PMC8907551; doi:10.12688/f1000research.52159.2)
Supplement: Peer review history

# Peer review: Reviewer response for version 2

**DOI:** 10.5256/f1000research.119241.r119403

**Published:** 2022-03-09

**Authors:** Ali Mobasheri

**Affiliations:** 1 Research Unit of Medical Imaging, Physics and Technology, Faculty of Medicine, University of Oulu, Oulu, Finland

I have looked at this paper and I have read the comments of the reviewers and authors. I am happy with this new version and I endorse it.

Is the work clearly and accurately presented and does it cite the current literature?

Yes

If applicable, is the statistical analysis and its interpretation appropriate?

Yes

Are all the source data underlying the results available to ensure full reproducibility?

Yes

Is the study design appropriate and is the work technically sound?

Yes

Are the conclusions drawn adequately supported by the results?

Yes

Are sufficient details of methods and analysis provided to allow replication by others?

Yes

Reviewer Expertise:

NA

I confirm that I have read this submission and believe that I have an appropriate level of expertise to confirm that it is of an acceptable scientific standard.

# Peer review: Reviewer response for version 1

**DOI:** 10.5256/f1000research.55397.r93903

**Published:** 2021-12-21

**Authors:** Anne-Christine Bay-Jensen

**Affiliations:** 1 ImmunoScience, Nordic Bioscience, Herlev, Denmark

This is a technical detailed manuscript on a well designed study. It is heavy to read, but informative. 

**Comments:**

- Were all biomarkers measurable in all subjects? If not, which value was given for the unmeasurable levels?
- Table 5: Does this really provide valuable information, as it is the same as figure 7 (if I understand it correctly).
- Figure 8: Is it necessary to show both figure 7 and 8 (correlations of relative vs. absolute change)? or could one of them be cited in the text? One of them seems enough to support the conclusion. 
- The reference is just a suggestion to the author of including the reference as part of the background information.‘The reference is just a suggestion to the author of including the reference as part of the background information.

Is the work clearly and accurately presented and does it cite the current literature?

Yes

If applicable, is the statistical analysis and its interpretation appropriate?

Yes

Are all the source data underlying the results available to ensure full reproducibility?

Yes

Is the study design appropriate and is the work technically sound?

Yes

Are the conclusions drawn adequately supported by the results?

Yes

Are sufficient details of methods and analysis provided to allow replication by others?

Yes

Reviewer Expertise:

Development and testing of biomarkers in healthy and rheumatology.

I confirm that I have read this submission and believe that I have an appropriate level of expertise to confirm that it is of an acceptable scientific standard.

## References

[1] : A biomarker perspective on the acute effect of exercise with and without impact on joint tissue turnover: an exploratory randomized cross-over study. *Eur J Appl Physiol* .2021;121(10) : 10.1007/s00421-021-04751-z 2799-2809 10.1007/s00421-021-04751-z 34156534

# Response

**Published:** 2021-12-22

**Authors:** Annegret Mündermann

**Affiliations:** University Hospital Basel, Switzerland

Dear Dr. Bay-Jensen,

 We would like to thank you for your feedback and your comments. We believe that the quality of our manuscript has improved by addressing the comments received. Please find our response to the comments below. Your comments by the reviewers are shown in quotation marks, our responses are shown below each comment in regular font.

- “Were all biomarkers measurable in all subjects? If not, which value was given for the unmeasurable levels?”
**Response: All biomarkers were measurable in all subject except ADAMTS-4 in subject 110. As described in the original manuscript, in this subject the values of ADAMTS-4 fluctuated much more than in all other participants and were outside the detection limits, and hence we excluded this subject from the analysis of ADAMTS-4.**
- “Table 5: Does this really provide valuable information, as it is the same as figure 7 (if I understand it correctly).”
**Response: The reviewer is correct that the Table and Figure contain some overlapping information. We now only report p-values in revised Table 5. Please note that the same point applies to Table 6 and Figure 8, and we have changed Table 6 accordingly. Please also note that the Table header of table 5 contained an error as absolute and not relative changes were correlated. We have corrected this error.**
- “Figure 8: Is it necessary to show both figure 7 and 8 (correlations of relative vs. absolute change)? or could one of them be cited in the text? One of them seems enough to support the conclusion.”
**Response: We agree that both Figures convey the same message. However, some previous studies report absolute values, and other report relative values. We believe that it is important to show that the results differ slightly whether one or the other is used but that the general pattern is the same. Hence, we think that it is important to show both Figures.**
- “The reference is just a suggestion to the author of including the reference as part of the background information.”
**Response: Thank you for providing this relevant and interesting reference that was published after our original manuscript was submitted. We now cite this reference in the introduction and discussion section.**

# Peer review: Reviewer response for version 1

**DOI:** 10.5256/f1000research.55397.r88074

**Published:** 2021-07-19

**Authors:** Ali Mobasheri

**Affiliations:** 1 Research Unit of Medical Imaging, Physics and Technology, Faculty of Medicine, University of Oulu, Oulu, Finland

**Overview:**

 In this article, the authors determined the suitability of a selected panel of biomarkers as mechanosensitive markers of articular cartilage and investigated the dose-response relationship between ambulatory load magnitude and marker kinetics in response to mechanical joint loading. They found that COMP, MMP-3, IL-6, MMP-9, and ADAMTS-4 respond to ambulatory load and they propose that these markers should be further investigated in the context of articular cartilage mechanosensitivity and its role in joint degeneration and osteoarthritis (OA).

**Major comments:**

 This is a well-written and comprehensive manuscript that should be of interest to the readership of F1000 Research. However, a number of important revisions are recommended.

  

- One of the major challenges associated with the use of commercially available enzyme-linked immunosorbent assays is that they measure the quantity of the protein to which the antibody within the assay was developed. Commercially available kits do not reflect important parameters such as epitopes and biological/enzymatic activity. For example, in the case of MMP-3 (stromelysin), the majority of commercially available kits do not measure active levels of MMP-3. Most of the commercial kits that are commercially available for MMP-3 measure total protein, but in most cases, only a small fraction of MMP-3 is proteolytically cleaved and activated. Therefore, all investigators need to carry out functional studies and estimate the quantity of active MMP-3. The use of commercially available enzyme-linked immunosorbent assays is widespread but unfortunately little biological and mechanistic insight is offered by kits that measure total protein levels. This results in numerous publications that simply talk about protein levels rather than epitopes and activity.
- The authors conclude that COMP, MMP-3, IL-6, MMP-9, and ADAMTS-4 are biomarkers that warrant further investigation in the context of articular cartilage mechanosensitivity and the role of cartilage mechanosensitivity in the development of OA.
- The observations that they have made in relation to COMP have been made by Lohmander, Strglics and others in Lund University in the context of joint injury. However key references are missing and have not been cited in this paper.
- The manuscript will benefit from an updated discussion of the role of protein activity (especially relating to matrix metalloproteinases) rather than total protein levels.
- A few missing references should be cited as well. (e.g. Dahlberg *et al.* (1994 ^1^), Lohmander *et al. *(1994 ^2^), Clutterbuck *et al. *(2011 ^3^)).

Is the work clearly and accurately presented and does it cite the current literature?

Yes

If applicable, is the statistical analysis and its interpretation appropriate?

Yes

Are all the source data underlying the results available to ensure full reproducibility?

Yes

Is the study design appropriate and is the work technically sound?

Yes

Are the conclusions drawn adequately supported by the results?

Yes

Are sufficient details of methods and analysis provided to allow replication by others?

Yes

Reviewer Expertise:

Biomarkers of osteoarthritis and articular cartilage turnover.

I confirm that I have read this submission and believe that I have an appropriate level of expertise to confirm that it is of an acceptable scientific standard, however I have significant reservations, as outlined above.

## References

[1] : Cartilage metabolism in the injured and uninjured knee of the same patient. *Ann Rheum Dis* .1994;53(12) : 10.1136/ard.53.12.823 823-7 10.1136/ard.53.12.823 7864691PMC1005483

[2] : Release of cartilage oligomeric matrix protein (COMP) into joint fluid after knee injury and in osteoarthritis. *Ann Rheum Dis* .1994;53(1) : 10.1136/ard.53.1.8 8-13 10.1136/ard.53.1.8 8311563PMC1005235

[3] : High throughput proteomic analysis of the secretome in an explant model of articular cartilage inflammation. *J Proteomics* .2011;74(5) : 10.1016/j.jprot.2011.02.017 704-15 10.1016/j.jprot.2011.02.017 21354348PMC3078332

# Response

**Published:** 2021-12-22

**Authors:** Annegret Mündermann

**Affiliations:** University Hospital Basel, Switzerland

Dear Dr. Mobasheri,

 We would like to thank you for your feedback and your critical comments. We believe that the quality of our manuscript has improved by addressing the comments received. Please find our response to the comments below. Your comments by the reviewers are shown in quotation marks, our responses are shown below each comment in regular font.

 “This is a well-written and comprehensive manuscript that should be of interest to the readership of F1000 Research.”

 Thank you.

 "However, a number of important revisions are recommended."

 We have addressed all comments as outlined below.

- “One of the major challenges associated with the use of commercially available enzyme-linked immunosorbent assays is that they measure the quantity of the protein to which the antibody within the assay was developed. Commercially available kits do not reflect important parameters such as epitopes and biological/enzymatic activity. For example, in the case of MMP-3 (stromelysin), the majority of commercially available kits do not measure active levels of MMP-3. Most of the commercial kits that are commercially available for MMP-3 measure total protein, but in most cases, only a small fraction of MMP-3 is proteolytically cleaved and activated. Therefore, all investigators need to carry out functional studies and estimate the quantity of active MMP-3. The use of commercially available enzyme-linked immunosorbent assays is widespread but unfortunately little biological and mechanistic insight is offered by kits that measure total protein levels. This results in numerous publications that simply talk about protein levels rather than epitopes and activity.”
**Response: The reviewer raises an important point.** **We have added this aspect as a limitation in the Discussion section and proposed that the current samples could be exploited in future analyses targeting epitopes and activity to offer further biological and mechanistic insight into the effect of well-defined mechanical stimuli on articular cartilage biology.**
- “The authors conclude that COMP, MMP-3, IL-6, MMP-9, and ADAMTS-4 are biomarkers that warrant further investigation in the context of articular cartilage mechanosensitivity and the role of cartilage mechanosensitivity in the development of OA.”
**No response is required.**
- “The observations that they have made in relation to COMP have been made by Lohmander, Strglics and others in Lund University in the context of joint injury. However key references are missing and have not been cited in this paper.”
**Response: We are aware of the previous studies on biomarkers in OA and injury and we now cite the stated reference in the introduction section. However, in our study, we focused on the dose-response relationship of load-induced blood marker concentrations and ambulatory load mechanics. No previous study has addressed this topic in patients with osteoarthritis or after injury. We will address this point in a currently ongoing trial.**
- “The manuscript will benefit from an updated discussion of the role of protein activity (especially relating to matrix metalloproteinases) rather than total protein levels.”
**Response: We agree that this is an important point. However, because the focus of our study was the dose-response relationship of load-induced blood marker concentrations and ambulatory load mechanics and we did not assess protein activity. Since we cannot provide data showing that protein activity depends on load magnitude, we believe that discussing this aspect is not within the scope of this manuscript. However, depending on available funding, we will pursue additional analyses on protein activity.**
- “A few missing references should be cited as well. (e.g. Dahlberg *et al.*(1994 ^1^ ), Lohmander *et al. *(1994 ^2^ ), Clutterbuck *et al. *(2011 ^3^ )).”
**Response: Thank you for providing these references. We have added these references in the context of biomarkers and OA respective joint injury in the Introduction section and in the context of the potential of future analyses using targeted or untargeted metabolomic and proteomic approaches in the Discussion section.**
